# Machine Learning Approaches for Predicting Progression to Alzheimer’s Disease in Patients with Mild Cognitive Impairment

**DOI:** 10.1007/s40846-024-00918-z

**Published:** 2024-12-24

**Authors:** Fatih Gelir, Taymaz Akan, Sait Alp, Emrah Gecili, Md. Shenuarin Bhuiyan, Elizabeth A. Disbrow, Steven A. Conrad, John A. Vanchiere, Christopher G. Kevil, The Alzheimer’s Disease Neuroimaging Initiative (ADNI), Mohammad Alfrad Nobel Bhuiyan

**Affiliations:** 1 Department of Medicine, Louisiana State University Health Sciences Center at Shreveport, PO Box 33932, Shreveport, LA 71103-4228 USA; 2 Department of Artificial Intelligence Engineering, Trabzon University, Trabzon, 61335 Turkey; 3 Division of Biostatistics and Epidemiology, Cincinnati Children’s Hospital Medical Center, Cincinnati, OH USA; 4 Department of Pediatrics, University of Cincinnati, Cincinnati, OH USA; 5 Department of Pathology and Translational Pathobiology, Louisiana State University Health Sciences Center at Shreveport, Shreveport, LA 71103 USA; 6 Department of Pharmacology, Louisiana State University Health Sciences Center at Shreveport, Toxicology & Neuroscience, Shreveport, LA 71103 USA; 7 Center for Brain Health, Louisiana State University Health Sciences Center at Shreveport, Shreveport, LA 71103 USA; 8 Department of Neurology, Louisiana State University Health Sciences Center at Shreveport, Shreveport, LA 71103 USA; 9 Department of Psychiatry, Louisiana State University Health Sciences Center at Shreveport, Shreveport, LA 71103 USA; 10 Department of Pediatrics, Louisiana State University Health Sciences Center at Shreveport, Shreveport, LA 71103 USA; 11 Department of Molecular and Cellular Physiology, Louisiana State University Health Sciences Center at Shreveport, Shreveport, LA 71103 USA

**Keywords:** Alzheimer's disease, Machine learning, Shapley value explanation technique, Feature selection, Balancing

## Abstract

**Purpose:**

Alzheimer's disease (AD), a neurodegenerative disorder, is a condition that impairs cognition, memory, and behavior. Mild cognitive impairment (MCI), a transitional stage before AD, urgently needs the development of prediction models for conversion from MCI to AD.

**Method:**

This study used machine learning methods to predict whether MCI subjects would develop AD, highlighting the importance of biomarkers (biological indicators from neuroimaging, such as MRI and PET scans, and molecular assays from cerebrospinal fluid or blood) and non-biomarker features in AD research and clinical practice. These indicators aid in early diagnosis, disease monitoring, and the development of potential treatments for MCI subjects. Using baseline data, which includes measurements of different biomarkers, we predicted disease progression at the patient’s last visit. The Shapley value explanation (SHAP) technique was used to identify key features for predicting patient progression.

**Results:**

The study used the ADNI database to evaluate the effectiveness of eight classification methods for predicting progression from MCI to AD. Four fundamental data sampling approaches were compared to balance the dataset and reduce overfitting. The SHAP technique improved the ability to identify biomarkers and non-biomarker features, enhancing the prediction of disease progression. NEAR-MISS was found to be the most advantageous sampling method, while XGBoost was found to be the superior classification method, offering enhanced accuracy and predictive power.

**Conclusion:**

The proposed SHAP for feature selection combined with XGBoost may provide improved predictive accuracy in diagnosing Alzheimer's patients.

## Introduction

In the United States, approximately 5.3 million individuals have Alzheimer's disease (AD), a number expected to rise to 13.8 million by 2050 [1]. AD currently ranks as the sixth most common cause of death in the United States [2]. It results in significant changes in the brain's structure and function. AD is a progressive brain disorder with a preclinical stage in which brain and blood changes are not currently measurable. It progresses to mild cognitive impairment (MCI) and finally to dementia, where memory, thinking, cognitive abilities, and behavioral symptoms impair daily life. AD is believed to begin at least 20 years before symptoms appear [3]. The transition from a healthy state to AD spans years, often starting with MCI before progressing to AD. However, not all individuals with MCI will develop AD.

Identifying stable MCI (sMCI) patients who do not progress to AD versus progressive MCI (pMCI) patients who are more likely to develop AD later is crucial [4]. Identifying pMCI patients enables early intervention strategies and potential treatments. Customizing clinical management based on progression likelihood optimizes patient care. Accurate identification is crucial for conducting clinical trials and providing patient and caregiver education, enabling informed decisions about healthcare and future support. In neurology and cognitive science, efforts have been ongoing to improve the accuracy of diagnosing and predicting MCI and develop biomarkers, neuroimaging techniques, and cognitive assessments to better distinguish between sMCI and pMCI and predict future outcomes.

Numerous studies focus on identifying AD biomarkers and non-biomarker features for preclinical research [5] using genetic, imaging, and biochemical methods to monitor progression and explore treatment options [6]. Alzheimer's databases have accelerated research by providing access to crucial data, fostering collaboration, and improving understanding of the disease's mechanisms and potential treatments. ADNI [7], launched in 2004 for $60 million, aims to evaluate the effectiveness of combining MRI, PET, biological markers, and clinical and neuropsychological assessments to measure the progression of mild cognitive impairment and early AD [8]. The initiative aims to identify sensitive features of early Alzheimer's disease progression, aiding researchers and clinicians in developing new treatments, monitoring effectiveness, and reducing the time and cost of clinical trials. It has optimized methods and standards for acquiring biomarker data [6]

Machine Learning (ML) is a crucial tool in medical research, aiding in patient classification, treatment prediction, and risk identification [9–11]. Over the past several years, ML techniques have gained attraction and are increasingly embraced and applied to AD analysis. In the past, computational neuroimaging studies have been conducted to predict the conversion of MCI patients to AD using various types of ADNI data. Gray et al. [12] used cross-sectional and longitudinal FDG-PET information for image-based classification of ADNI participants. Using a Support Vector Machine (SVM) classifier, the study classified four diagnostic groups and identified regional features with an accuracy of 63.1%. Young et al. [13] introduced the Gaussian process (GP) classification, a Bayesian method that produced probabilistic predictions, which correlated well with conversion to AD within 3 years in 96 sMCI and 47 pMCI subjects, with a balanced accuracy of 74%. Casanova et al. [14] used regularized logistic regression (RLR) and MRI data, achieving an accuracy of 61.5% in distinguishing between pMCI and sMCI after a rigorous tenfold cross-validation process. Salvatore et al. [15] introduced a machine-learning classification algorithm using SVM to identify discriminative features in the MRI images of AD patients. The algorithm achieved a classification accuracy of 66% for distinguishing between MCI and AD. Ritter et al. [16] compared three advanced classification algorithms, SVM, a single classification tree, and Random Forests, focusing on predicting the progression from MCI to AD. The study achieved 73% accuracy with SVM, evaluating feature importance across various sets and comparing automatic and manual feature selection methods. Abrol et al. [17] used a modified deep residual neural network (ResNet) to predict the progression from MCI to AD, with a mean test accuracy of 75.1%. Gao et al*.* [18] proposed an age-adjust neural network (AD-NET) with the pre-training model to predict MCI-to-AD conversion. The study used fivefold cross-validation on the ADNI cohort, resulting in an accuracy rate of 76%. To improve classification performance, Xiao et al. [19] used sparse logistic regression (SLR) to select informative brain regions from neuroimaging data. The study assessed 197 subjects from the ADNI database, achieving a classification accuracy of 72.87% using SLR in pMCI vs. sMCI.

Various ML methods have been applied to classify stages of AD with varying success. Support Vector Machines, Gaussian processes, and neural networks such as ResNet and AD-NET have been used the most, demonstrating accuracies from the low 60 s to approximately 76%. These studies have used both FDG-PET and MRI imaging data to identify diagnostic features and predict disease progression, highlighting the potential of ML in enhancing diagnostic precision in AD research.

The challenge of comparing studies in AD research arises from the lack of standardized datasets and training protocols. While ADNI provides valuable data, researchers may select data based on personal preferences, leading to unequal sample sizes and non-overlapping datasets across studies. This variability complicates the comparison of method accuracy between different models trained on disparate datasets. Our study adopts a rigorous approach to address this challenge by conducting evaluations using consistent datasets and methodologies. We employed eight classification methods and three feature selection techniques, ensuring all models were tested on the same set of samples. This approach eliminates biases introduced by dataset variations, allowing for a more equitable comparison of predictive models for the progression from MCI to AD.

Moreover, AD biomarker data is variable due to heterogeneous etiology, causing challenges in diagnosis and understanding [20]. Variations in acquisition protocols, multicentric study designs, and MRI preprocessing errors complicate the identification and interpretation of biomarkers, affecting their sensitivity and specificity in detecting AD-related changes. Addressing these issues improves biomarker reliability and accuracy.

The Shapley value explanation (SHAP) technique is a concept found in cooperative game theory that distributes the payoff among players based on their contribution to the game. In ML, SHAP values provide a consistent and accurate explanation for model output by attributing the prediction output to each input feature's contribution. This enables model interpretability, helping to determine which features are most influential in the model's predictions. SHAP offers transparency in predictive modeling, which is crucial for clinical decision-making and research in complex domains such as healthcare, particularly AD classification. By elucidating the most influential biomarkers in diagnosing AD, researchers and clinicians can better understand the disease's underlying mechanisms and tailor interventions more precisely. SHAP values can provide a robust framework for identifying and interpreting relevant biomarkers, improving the sensitivity and specificity of biomarkers in detecting AD-related changes. This enables the adoption of AI tools in healthcare, fostering trust and bridging the gap between complex ML models and practical clinical applications.

In this study, we were motivated by the critical role of feature importance in addressing the complexities of AD research. Given the disease's heterogeneity and variable patterns, identifying key biomarkers or imaging features is crucial for better understanding and developing practical diagnostic tools. By prioritizing the most relevant features, we propose identifying the most prevalent biomarkers indicating conversion from MCI to AD. Furthermore, emphasizing the importance of features, we aim to reduce the effects of data variability, allowing researchers to retain relevant information while minimizing noise or artifacts in AD research.

## Materials and Methods

### AD Data

The data used in this article are sourced from a database available at adni.loni.ucla.edu. ADNI aims to integrate serial MRI, PET, biological markers, and clinical assessments to measure the progression of MCI and early AD. In our study, we used baseline data as the primary input features. The associated features were derived from the ADNI database processed by the University of California, San Francisco (UCSF) team using FreeSurfer (http://surfer.nmr.mgh.harvard.edu/), involving cortical reconstruction and volumetric segmentations. These MRI features were sourced from 819 subjects (188 AD, 402 MCI, and 229 Normal). From the initial 819 subjects, we extracted 402 subjects over 6 to 186 months labeled as MCI at baseline. MCI patients were observed over time, and, based on their progression, labels were assigned to them as either 0 or 1. Patients who did not develop AD were labeled 0, while those who did develop AD were labeled 1. Additionally, among these 402 MCI subjects, 13 transitioned to Cognitive normal (CN), 172 remained in the MCI stage, and 217 progressed to AD. In the merged dataset, we had 47 columns, attached as a Supplementary Excel file (Features.xlsx), extracting the 20 most important features based on the feature selection method.

### Method and Data Management

We first use a preprocessing step consisting of streamlining the information, eliminating redundancies, and handling missing values effectively. First visit information was retained, while all other information was excluded, except for the last visit and the diagnosis recorded. Redundant, unrelated, or collinear features were removed to enhance clarity and reduce complexity. Baseline integrity preservation was maintained by retaining all features from the baseline visit. Missing values were imputed using a K-Nearest Neighbors (KNN) method, with the five nearest neighbors contributing to a robust strategy. Scaling ensures uniformity and mitigates the impact of varying scales among different features. This comprehensive data preprocessing strategy transforms all values into a standardized range between 0 and 1, facilitating equally weighted comparisons and preventing certain features from disproportionately influencing analyses. The dataset was systematically refined, removing irrelevant information, handling missing values, and standardizing feature scales.

Initially, the dataset contained several features deemed irrelevant for our analysis due to their nature, such as IMAGEUID, Site, and Subject ID, which do not contribute to the predictive modeling of disease progression. These features were removed to ensure the focus remained on clinically relevant biomarkers. Additionally, certain features had a substantial proportion of missing values. Given the extent of missing data and the difficulty in accurately imputing these values without introducing bias, those features were excluded from our analysis. Moreover, to maintain consistency and control for temporal variability, we restricted our feature set to include only baseline data (bl suffix in feature names), discarding features related to subsequent visits. This approach was taken to isolate the initial clinical state of the subjects, which is crucial for our study's focus on early prediction of AD progression. These preparation steps created a more robust and informative dataset for meaningful explorations and modeling.

Second, we used feature selection algorithms separately to find biomarkers to build robust learning models. Clinical research indicates that critical biomarkers in AD are essential to providing diagnostically relevant information, particularly in early disease stages, as they reflect key elements of AD pathophysiology [21]. Therefore, we aimed to determine the most significant biomarkers. We examined three top-performing, cutting-edge feature selection algorithms: SHAP [22], the Gini index [23], and Extreme Gradient Boosting (XGBoost) [24] for relevant feature identification. In addition, we also focused on the standard features identified by these methods.

Next, we compared four fundamental data sampling approaches to balance the groups in the dataset and mitigate overfitting. A no-sampling approach was also considered. It is worth mentioning that external methods for addressing class imbalances are highly adaptable and operate independently of specific feature selection or classification algorithms. The data sampling approaches investigated in this study include no-sampling, random under-sampling, random oversampling, synthetic minority over-sampling technique (SMOTE) oversampling, and NEAR-MISS sampling. No-sampling contains all data points from majority and minority training sets. Random under-sampling includes all minority class training data points, eliminating instances from the majority until the desired balance is achieved. This method has the drawback of potentially losing valuable information from the majority class due to under-sampling. Random oversampling includes all data points from both sets and randomly selects instances from the minority training set until the desired balance is achieved. Repeated additions of the same minority samples may lead to overfitting, diminishing the classifier's generalization ability. SMOTE oversampling generates new synthetic data by randomly interpolating pairs of nearest neighbors. This study uses SMOTE exclusively for oversampling and will be labeled SMOTE in subsequent tables and figures. NEAR-MISS sampling is a ML under-sampling technique that aims to reduce the number of instances from the majority class by selecting ‘close’ samples close to those in the minority class.

Finally, our classification process involved the implementation of eight classifiers: Random Forest (RF) [25], Support Vector Machine (SVM) [26], K-Nearest Neighbors (KNN) [27], Logistic Regression [28], Decision Trees [29], XGBoost, Gradient Boosting [30], and Artificial Neural Networks (ANNs) [31] leveraging the top-selected features.

## Results

We report on the potential of specific biomarkers to predict the transition from MCI to AD. We conducted this study to evaluate biomarkers acquired from MRI scans using FreeSurfer to reveal their potential in predicting AD progression. This section describes the results of the experiments based on SHAP, XGBoost, and the Gini index feature selection methods alongside various sampling and classification methods. The sampling methods used include Random Over Sampling (ROS), Random Under Sampling (RUS), SMOTE, NEAR-MISS, and No Sampling. In contrast, the classification methods comprise Support Vector Machines (SVM), Logistic Regression (LR), K-Nearest Neighbors (KNN), Artificial Neural Networks (ANNs), Gradient Boosting (GB), XGBoost, Random Forest (RF), and Decision Tree (DT).

This study's initial dataset comprised 7,240 observations and 57 variables, capturing a wide range of patient measurements and baseline information. We prioritized the analysis of baseline variables alongside the labels obtained from the last visit; consequently, the total observations were reduced to 819 unique subjects. Due to a significant number of missing values in certain variables, the dataset underwent a refinement process, reducing the number of variables from 57 to 47. Subsequently, by applying three distinct feature selection algorithms—SHAP, the Gini index, and XGBoost—we identified and retained the top 20 most significant features. Our analysis was further specialized by selecting only those observations with an MCI baseline, culminating in a dataset of 402 observations. Prior to implementing sampling techniques, this dataset was partitioned via a tenfold cross-validation approach, resulting in a training set of 362 samples and a fixed test set comprising 40 samples. The application of oversampling methods, specifically SMOTE and ROS, expanded our training set to 390 samples. Conversely, under-sampling techniques such as NEAR-MISS and RUS reduced the training sample size to 334, ensuring a more balanced and representative dataset for subsequent analyses.

We used the K-fold cross-validation protocol for training and testing, with K set to 10. K-fold cross-validation reduces overfitting to training data. The optimal parameter settings for ML algorithms are presented in Table 1, ensuring robustness in the ML workflow. The optimal parameters are highlighted in bold, providing a detailed overview of the methodological framework adopted in this study.

**Table 1:** The parameter settings for each algorithm, with the optimal parameters identified through evaluation highlighted in bold

Classifier	Parameters	Optimized Values
KNN	p	[**Manhattan distance**, Euclidean distance]
	k	[1, 3, 5, 9, 11]
LR	Max iteration	1000
	Tolerance	5e-4
	Solver	Lib-linear
	C	[0.001, 0.01, 0.1, **1**, 10, 100, 1000]
SVM	Probability	True
	Kernel	Radial Basis Function (RBF)
	C	[0.1, **1**, 10]
	gamma	[0.01, 0.1, **1**]
DT	criterion	Gini
	Depth	[2, 4, 6, 8, 10, 12, 14]
RF	N estimators	[50, 100, 200, **500**,1000]
	criterion	Gini
	Max features	[**sqrt**, log2]
	Min-samples -leaf	[1, 3]
GB	N estimators	[50, 100, 200, 500]
	Max leaf nodes	[2, 4, 8, 16]
	Learning rate	[1, 0.5, **0.1**, 0.05, 0.01]
XGB	Max depth	[3, 5, 7, 9]
	Min child weight	[1, 3]
	Gamma	[0, **0.1**, 0.2]
	Subsample	[0.6, **0.8**]
	Col sample by tree	[**0.6**, 0.8]
	Reg alpha	[0, 0.005, 0.01, **0.05**]
ANN	Hidden layer sizes	[20, (20, 10), 50, (50, 20), **100**]
	Activation	[**relu**, logistic]
	L2 regularization	[0.0001, 0.001, **0.09**, 0.1, 0.5]
	Optimizer	[**Adam**, SGD]
	Loss	Cross-Entropy

KNN = K-Nearest Neighbors, LR = Logistic Regression, SVM = Support Vector Machine, DT = Decision Tree, RF = Random Forest, GB = Gradient Boosting, XGB = XGBoost, and ANN = Artificial Neural Networks

**Fig. 1:**
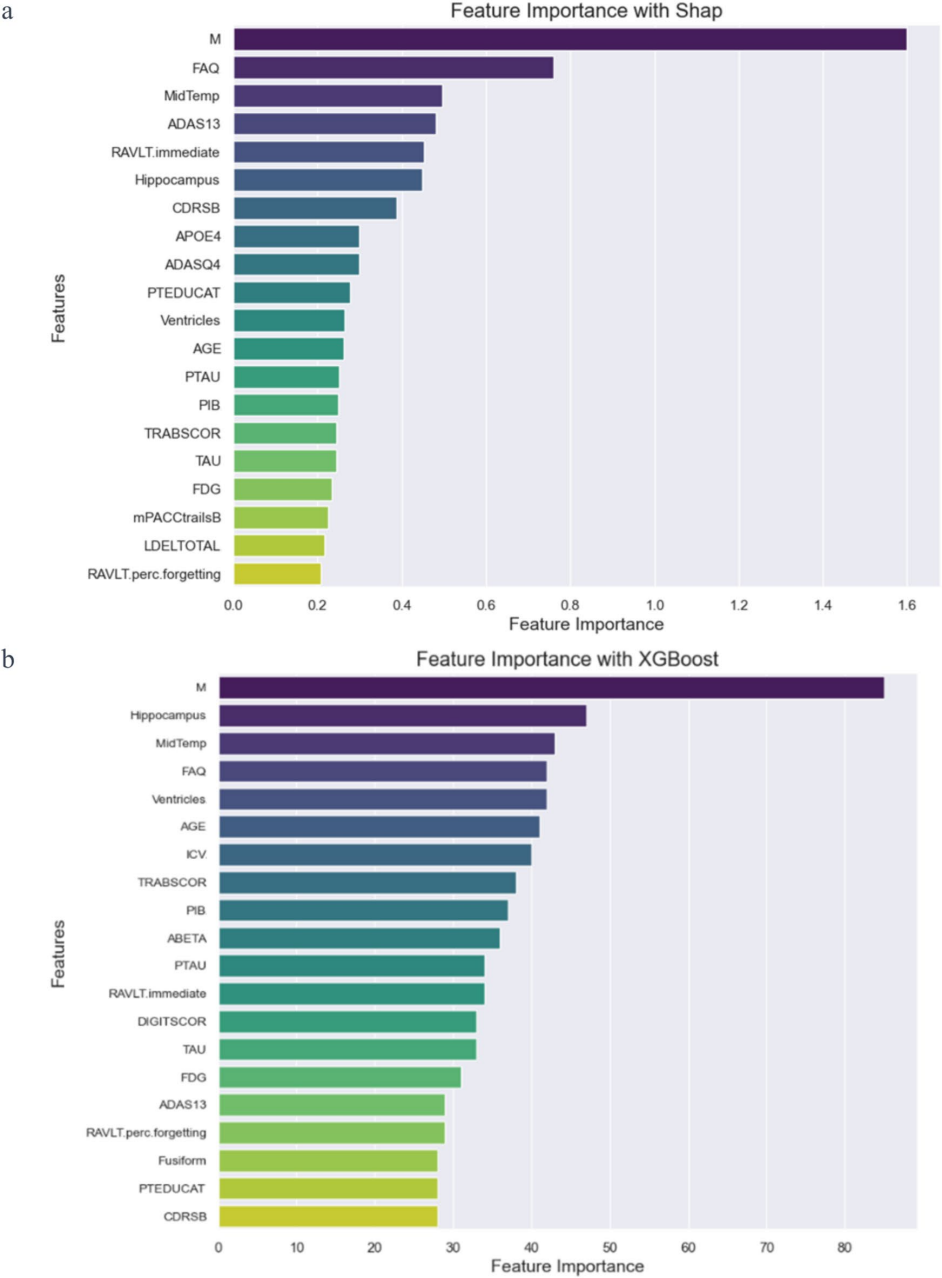

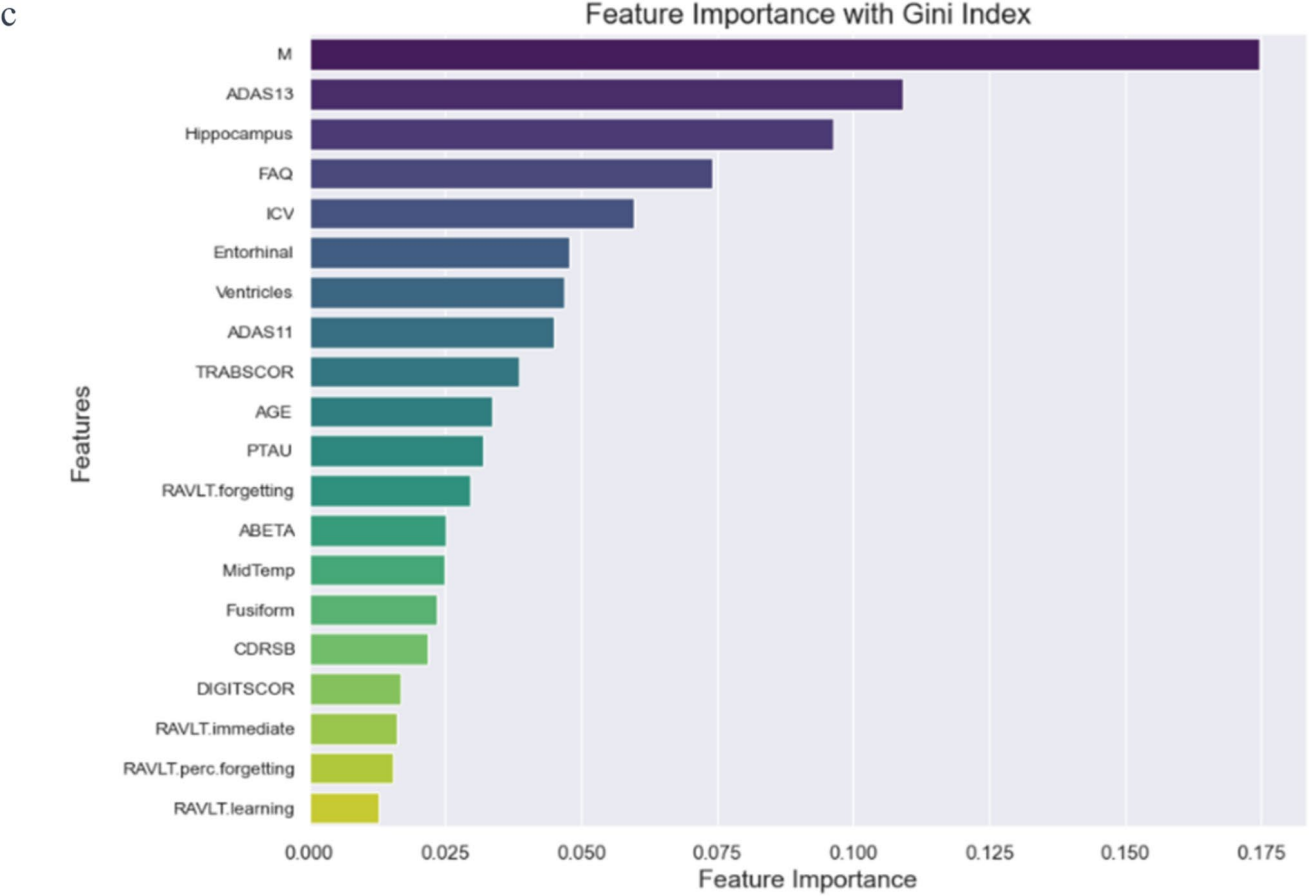
Comparative visualization of feature importance across SHAP **a**, XGBoost **b**, and the Gini index **c** methods

Figure 1 illustrates the top 20 features (M = Month Since Baseline; RAVLT = Rey Auditory Verbal Learning Test immediate (sum of 5 trials); CDR-SB = The Clinical Dementia Rating Scale; APOE4 = APOEe4 alleles; ADASQ4 = ADAS Delayed Word Recall; PTEDUCAT = Education; PTAU= Phosphorylated tau protein (p-tau) in cerebrospinal fluid; PIB = Pittsburgh Compound B for quantitative measure of amyloid beta plaque; TRABSCOR = Trail Making Test- Part B; TAU the measurement of “total tau” protein levels in cerebrospinal fluid ; FDG= brain metabolism using Positron Emission Tomography with the tracer Fluorodeoxyglucose, for specific regions of interest (ROIs); mPACCtrailsB = modified Preclinical Alzheimer's Cognitive Composite; LDELTOTAL = Logical Memory - Delayed Recall; RAVLT.perc.forgetting = Rey Auditory Verbal Learning Test percent forgetting;ICV = Intracranial Volume; ABETA = Measurement of amyloid beta protein;DIGITSCOR = Digit Symbol Substitution Score;Fusiform = fusiform gyrus;Entorhinal = entorhinal cortex; RAVLT.forgetting = Rey Auditory Verbal Learning Test (trial 5 - delayed) forgetting score; RAVLT.learning = Rey Auditory Verbal Learning Test (trial 5 – Trial 1) learning score) crucial for predicting cognitive impairment progression through three distinct feature selection methods: the Gini index, XGBoost, and SHAP. The ‘. bl’ suffix in the variable names indicates that the data represent baseline measurements, capturing the initial state of the subjects at the start of the study, prior to any interventions or further progression of conditions. These methods identified 12 common features, including age and specific cognitive assessment scores, as key biomarkers. However, features such as Fusiform ('Fusiform.bl') and entorhinal ('Entorhinal.bl') at baseline showed varied importance levels, highlighting the distinct methodological focuses of the Gini index's reliance on impurity reduction, XGBoost's gradient boosting for feature prioritization, and SHAP's emphasis on interpretability and individual feature contributions. The variability in their importance highlights each method's nuanced approach to feature selection and the complexity of AD predictive modeling. The intersection of these methods provides a solid foundation for more robust predictive modeling, but the unique attributes identified by each method can provide valuable insights. Integrating the strengths of the Gini index, XGBoost, and SHAP reaffirms the most critical biomarkers and the synergistic power of combined feature selection approaches, suggesting a pathway toward a more comprehensive and nuanced predictive model (Table 2).

**Table 2:** Biomarkers selected by SHAP, XGBoost, the Gini index, and those identified as common features across methods for the prediction of conversion from MCI to AD

	SHAP	XGboost	Gini index	Common feature
M	✓	✓	✓	✓
MidTemp	✓	✓	✓	✓
ADAS13	✓	✓	✓	✓
RAVLT Immediate	✓	✓	✓	✓
Hippocampus	✓	✓	✓	✓
CDR-SB	✓	✓	✓	✓
APOE4	✓			
ADASQ4	✓			
PTEDUCAT	✓	✓		
Ventricles	✓	✓	✓	✓
AGE	✓	✓	✓	✓
PTAU	✓	✓	✓	✓
PIB	✓	✓		
TRABSCOR	✓	✓	✓	✓
TAU	✓	✓		
FDG	✓	✓		
mPACCtrailsB	✓			
LDELTOTAL	✓			
RAVLT.perc.forgetting	✓	✓	✓	✓
ICV		✓	✓	
ABETA		✓	✓	
DIGITSCOR		✓	✓	
Fusiform		✓	✓	
Entorhinal			✓	
ADAS11			✓	
RAVLT.forgetting			✓	
RAVLT.learning			✓	

M = Month Since Baseline; RAVLT = Rey Auditory Verbal Learning Test immediate (sum of 5 trials); CDR-SB = The Clinical Dementia Rating Scale; APOE4 = APOEe4 alleles; ADASQ4 = ADAS Delayed Word Recall; PTEDUCAT = Education; PTAU = Phosphorylated tau protein (p-tau) in cerebrospinal fluid; PIB = Pittsburgh Compound B for quantitative measure of amyloid beta plaque; TRABSCOR = Trail Making Test- Part B; TAU the measurement of "total tau" protein levels in cerebrospinal fluid; FDG = brain metabolism using Positron Emission Tomography with the tracer Fluorodeoxyglucose, for specific regions of interest (ROIs); mPACCtrailsB = modified Preclinical Alzheimer's Cognitive Composite; LDELTOTAL = Logical Memory—Delayed Recall; RAVLT.perc.forgetting = Rey Auditory Verbal Learning Test percent forgetting;ICV = Intracranial Volume; ABETA = Measurement of amyloid beta protein;DIGITSCOR = Digit Symbol Substitution Score;Fusiform = **fusiform gyrus;**Entorhinal = **entorhinal cortex;**; RAVLT.forgetting = Rey Auditory Verbal Learning Test (trial 5—delayed) forgetting score; RAVLT.learning = Rey Auditory Verbal Learning Test (trial 5 – Trial 1) learning score

Tables 3, 4, and 5 display classification results using different feature selection approaches and sampling and classification methods.

**Table 3:** Classification results using SHAP feature selection to explore various sampling and classification methods for optimal model performance

Sampling Method	Model	Accuracy	Avg Precision	Recall	F1 Score
ROS	SVM	0.7166	0.8446	0.7284	0.7346
ROS	LG	0.7141	0.8298	0.719	0.7292
ROS	KNN	0.6892	0.8097	0.7052	0.7091
ROS	ANNs	0.7214	0.8683	0.7372	0.7409
ROS	GB	0.7492	0.8552	0.7567	0.7628
ROS	XGB	0.7465	0.8778	0.7797	0.766
ROS	RF	0.7589	0.8536	0.7654	0.7735
ROS	DT	0.6593	0.6782	0.6835	0.6817
No Sampling	SVM	0.7388	0.8561	0.7424	0.7543
No Sampling	LG	0.7264	0.8344	0.7281	0.7405
No Sampling	KNN	0.7216	0.814	0.7929	0.7552
No Sampling	ANNs	0.7236	0.8592	0.7554	0.7463
No Sampling	GB	0.7215	0.8446	0.7563	0.745
No Sampling	XGB	0.759	0.8847	0.7981	0.7792
No Sampling	RF	0.7488	0.8446	0.7883	0.7709
No Sampling	DT	0.6565	0.698	0.7333	0.6976
SMOTE	SVM	0.7064	0.827	0.7374	0.7307
SMOTE	LG	0.7215	0.835	0.7193	0.7335
SMOTE	KNN	0.694	0.8005	0.6968	0.709
SMOTE	ANNs	0.7088	0.8593	0.7193	0.7265
SMOTE	GB	0.7217	0.8403	0.7195	0.7331
SMOTE	XGB	0.7415	0.8614	0.7429	0.7539
SMOTE	RF	0.7513	0.8449	0.7797	0.7713
SMOTE	DT	0.6566	0.6771	0.6918	0.6813
RUS	SVM	0.7314	0.849	0.7331	0.7468
RUS	LG	0.7239	0.8321	0.7284	0.7386
RUS	KNN	0.6967	0.8181	0.6959	0.7105
RUS	ANNs	0.7115	0.816	0.7143	0.7252
RUS	GB	0.7365	0.8505	0.719	0.7463
RUS	XGB	0.7491	0.8801	0.7558	0.7637
RUS	RF	0.7563	0.8545	0.761	0.7704
RUS	DT	0.6841	0.7088	0.7424	0.7139
NEAR-MISS	SVM	0.7165	0.8375	0.7697	0.7459
NEAR-MISS	LG	0.7066	0.8359	0.7377	0.7296
NEAR-MISS	KNN	0.724	0.8116	0.7838	0.7543
NEAR-MISS	ANNs	0.6766	0.8304	0.7052	0.7005
NEAR-MISS	GB	0.7437	0.8695	0.7567	0.7608
NEAR-MISS	XGB	0.7538	0.8645	0.747	0.7638
NEAR-MISS	RF	0.7613	0.8627	0.7567	0.7722
NEAR-MISS	DT	0.6718	0.7187	0.6877	0.6909

KNN = K-Nearest Neighbors, LR = Logistic Regression, SVM = Support Vector Machine, DT = Decision Tree, RF = Random Forest, GB = Gradient Boosting, XGB = XGBoost, and ANN = Artificial Neural Networks

**Table 4:** Classification results using XGBoost feature selection to explore various sampling and classification methods for optimal model performance

Sampling Method	Model	Accuracy	Avg Precision	Recall	F1 Score
ROS	SVM	0.7362	0.8501	0.7374	0.7498
ROS	LG	0.689	0.8023	0.6918	0.7051
ROS	KNN	0.6346	0.7776	0.6732	0.6643
ROS	ANNs	0.7142	0.8506	0.7281	0.7316
ROS	GB	0.714	0.8336	0.7292	0.7297
ROS	XGB	0.7439	0.8624	0.7613	0.7586
ROS	RF	0.7415	0.8451	0.747	0.7555
ROS	DT	0.6541	0.6931	0.6502	0.6673
No Sampling	SVM	0.7387	0.855	0.7515	0.7551
No Sampling	LG	0.6916	0.8117	0.7011	0.7097
No Sampling	KNN	0.672	0.7769	0.7838	0.721
No Sampling	ANNs	0.7241	0.8511	0.7513	0.7437
No Sampling	GB	0.7241	0.8345	0.7563	0.7464
No Sampling	XGB	0.7415	0.8524	0.7513	0.7544
No Sampling	RF	0.739	0.8482	0.7801	0.7627
No Sampling	DT	0.6665	0.7003	0.6963	0.694
SMOTE	SVM	0.7413	0.8527	0.761	0.7587
SMOTE	LG	0.689	0.8095	0.7011	0.7079
SMOTE	KNN	0.6541	0.7647	0.6868	0.6824
SMOTE	ANNs	0.7166	0.8563	0.7281	0.7334
SMOTE	GB	0.7165	0.8401	0.7333	0.735
SMOTE	XGB	0.7563	0.8622	0.7658	0.7669
SMOTE	RF	0.7415	0.8457	0.7377	0.7535
SMOTE	DT	0.6341	0.6679	0.6504	0.6537
RUS	SVM	0.7388	0.843	0.7513	0.7559
RUS	LG	0.6866	0.8067	0.7009	0.7069
RUS	KNN	0.6444	0.7668	0.6864	0.6749
RUS	ANNs	0.7142	0.8416	0.742	0.7338
RUS	GB	0.7015	0.8502	0.6773	0.7065
RUS	XGB	0.7463	0.8467	0.7329	0.7559
RUS	RF	0.7266	0.8362	0.7199	0.7392
RUS	DT	0.6741	0.698	0.7058	0.696
NEAR-MISS	SVM	0.7438	0.8554	0.7606	0.7606
NEAR-MISS	LG	0.6915	0.8186	0.7193	0.7193
NEAR-MISS	KNN	0.6745	0.7737	0.7747	0.7747
NEAR-MISS	ANNs	0.7241	0.8582	0.742	0.742
NEAR-MISS	GB	0.7365	0.8544	0.7522	0.7522
NEAR-MISS	XGB	0.7241	0.8356	0.7279	0.7279
NEAR-MISS	RF	0.734	0.8362	0.7238	0.7238
NEAR-MISS	DT	0.6666	0.6995	0.6916	0.6916

KNN = K-Nearest Neighbors, LR = Logistic Regression, SVM = Support Vector Machine, DT = Decision Tree, RF = Random Forest, GB = Gradient Boosting, XGB = XGBoost, and ANN = Artificial Neural Networks

**Table 5:** Classification results using the Gini index feature selection to explore various sampling and classification methods for optimal model performance

Sampling Method	Model	Accuracy	Avg Precision	Recall	F1 Score
ROS	SVM	0.7016	0.8312	0.7149	0.7205
ROS	LG	0.6693	0.7966	0.6779	0.6884
ROS	KNN	0.6668	0.7945	0.7236	0.6996
ROS	ANNs	0.7116	0.851	0.71	0.7241
ROS	GB	0.7216	0.8566	0.7426	0.7394
ROS	XGB	0.749	0.8397	0.7742	0.7661
ROS	RF	0.724	0.8393	0.7331	0.7413
ROS	DT	0.622	0.6515	0.6372	0.6414
No Sampling	SVM	0.7115	0.8325	0.7102	0.7257
No Sampling	LG	0.6742	0.8008	0.6918	0.6958
No Sampling	KNN	0.6668	0.7915	0.7701	0.7108
No Sampling	ANNs	0.7313	0.849	0.7654	0.7522
No Sampling	GB	0.7464	0.8599	0.8028	0.7722
No Sampling	XGB	0.7193	0.8409	0.7424	0.7402
No Sampling	RF	0.729	0.8377	0.7758	0.7538
No Sampling	DT	0.6342	0.6732	0.6736	0.6655
SMOTE	SVM	0.719	0.8299	0.7333	0.7369
SMOTE	LG	0.6817	0.7961	0.6872	0.699
SMOTE	KNN	0.6866	0.7845	0.71	0.7074
SMOTE	ANNs	0.7166	0.8458	0.7152	0.7282
SMOTE	GB	0.7364	0.8575	0.7468	0.752
SMOTE	XGB	0.7315	0.8571	0.7519	0.749
SMOTE	RF	0.724	0.8395	0.7381	0.7418
SMOTE	DT	0.6363	0.6508	0.6312	0.6467
RUS	SVM	0.6941	0.8249	0.6963	0.7092
RUS	LG	0.6817	0.7912	0.6916	0.7012
RUS	KNN	0.6595	0.7847	0.7056	0.6885
RUS	ANNs	0.7191	0.8266	0.7333	0.7353
RUS	GB	0.729	0.8579	0.747	0.7473
RUS	XGB	0.7243	0.8276	0.7006	0.7296
RUS	RF	0.724	0.8389	0.7247	0.7376
RUS	DT	0.6393	0.6727	0.6736	0.6671
NEAR-MISS	SVM	0.7015	0.8294	0.7011	0.7151
NEAR-MISS	LG	0.6816	0.8099	0.7193	0.71
NEAR-MISS	KNN	0.6718	0.784	0.7701	0.7143
NEAR-MISS	ANNs	0.7067	0.8382	0.7279	0.725
NEAR-MISS	GB	0.7288	0.8577	0.7377	0.7436
NEAR-MISS	XGB	0.7465	0.8471	0.7513	0.7577
NEAR-MISS	RF	0.7389	0.8472	0.7245	0.7488
NEAR-MISS	DT	0.6419	0.6825	0.674	0.6703

KNN = K-Nearest Neighbors, LR = Logistic Regression, SVM = Support Vector Machine, DT = Decision Tree, RF = Random Forest, GB = Gradient Boosting, XGB = XGBoost, and ANN = Artificial Neural Networks

Table 3 reports the classification results of using SHAP feature selection alongside various sampling and classification methods. The table reveals intriguing insights into the performance of different combinations. Among the sampling techniques investigated, the NEAR-MISS method consistently demonstrated robust performance across multiple classification models, mainly showcasing substantial accuracy and average precision. Notably, models trained on NEAR-MISS exhibited the highest accuracy values compared to other sampling methods, with the highest achieved accuracy of 0.7613 obtained with RF. The RUS technique also showed competitive results, particularly in recall and F1 scores. Regarding classification algorithms, the XGBoost method stood out, consistently achieving high accuracy, average precision, recall, and F1 scores across different sampling methodologies. These findings suggest that a combination of NEAR-MISS sampling with XGBoost classification could yield the most favorable results in this scenario.

**Fig. 2:**
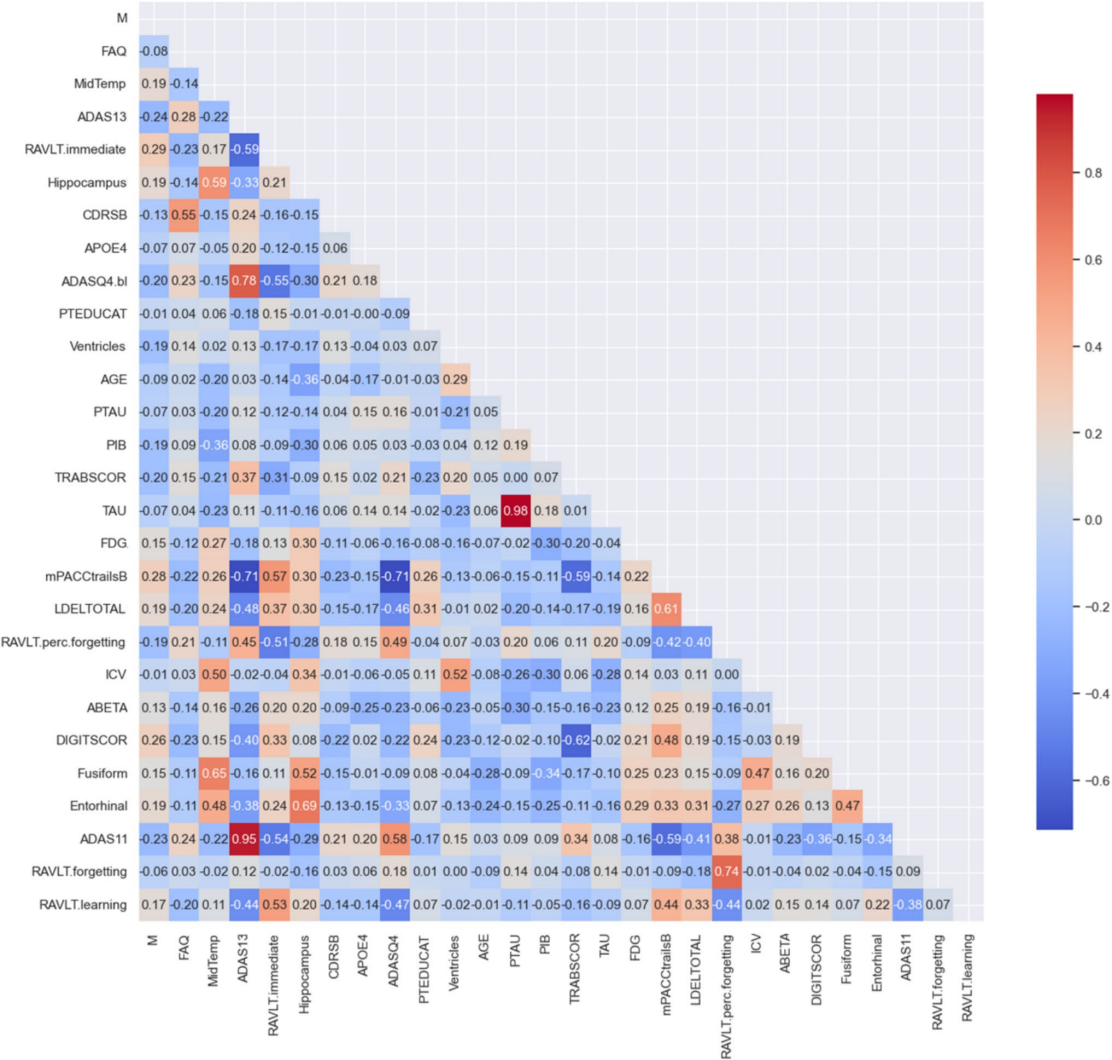
The correlation matrix of the union of features selected by three different feature selection methods (SHAP, XGBoost, and the Gini index) is used to identify key biomarkers for predicting the transition from MCI to AD

Figure 2 displays the correlation matrix for the features identified through the union of three feature selection methods. This visualization illustrates the interrelationships among these critical variables, underscoring potential areas for further investigation based on the strength and nature of their correlations.

Our analysis employed machine learning methods to identify key biomarkers predicting the transition from MCI to AD. Table 2 summarizes the biomarkers selected by each method—SHAP, XGBoost, and the Gini index—and those commonly identified by all three methods. This comparative overview aids in understanding the relative importance and consensus of predictive features across different analytical approaches. This correlation matrix visually represents the pairwise correlations between various biomarkers, as indicated in our research study. The cells in the matrix show correlation coefficients ranging from -1 to 1, represented by a color gradient from blue (negative correlation) to red (positive correlation). Notable high correlations can be observed between several pairs, such as PTAU and TAU (0.98), ADAS11 and ADAS13 (0.95), ADASQ4 and ADAS13 (0.78), and RAVLT.immediate and RAVLT.learning (0.53).

Our study's biomarkers and non-biomarker features comprehensively address various aspects of AD, offering insights into its progression, symptoms, and underlying mechanisms. Duration of study participation (M) is pivotal for tracking disease progression over time. Functional abilities are assessed through the Functional Activities Questionnaire (FAQ), which helps gauge the cognitive impairment severity typical in AD. The Middle Temporal Area (MidTemp) imaging detects atrophy, a trait common to AD progression. The severity of cognitive symptoms is quantified using the ADAS13 scale, while the Rey Auditory Verbal Learning Test (RAVLT.immediate) measures impaired short-term auditory-verbal memory, an early sign in AD cases. Hippocampal volume loss, a hallmark of AD, correlates with memory decline and is assessed further using the Clinical Dementia Rating Scale-B (CDRSB). The genetic risk factor APOE4 and psychotic symptoms (ADASQ4) contribute to understanding genetic predispositions and behavioral manifestations. Educational levels (PTEDUCAT) indicate a potential cognitive reserve that might delay dementia onset, and ventricular enlargement reflects brain tissue degeneration. Critical biomarkers include PTAU and TAU, indicating neurofibrillary tangles and neuronal damage through elevated cerebrospinal fluid levels. Pittsburgh Compound B (PIB) and Fluorodeoxyglucose (FDG) are used in PET scans to detect amyloid plaques and areas of reduced glucose metabolism, respectively. The Modified Preclinical Alzheimer Cognitive Composite (mPACCtrailsB) and various RAVLT measures (immediate, percent forgetting, forgetting, and learning) highlight cognitive changes, memory impairment, and learning abilities in AD patients. In addition, the Intracranial Volume (ICV) controls brain imaging to adjust for individual brain size differences, while ABETA levels in CSF indicate amyloid deposition. The Digit Symbol Substitution Test (DIGITSCOR) assesses cognitive functions such as processing speed and memory, and imaging studies of the fusiform and entorhinal regions help predict AD onset by identifying areas affected early in the disease process.

Table 3 presents the classification results achieved using different sampling methods and classification algorithms, with feature selection performed using the SHAP feature selection method. Notably, the combination of NEAR-MISS with RF classification attained the highest accuracy of 0.7613. Conversely, combining SMOTE with DT classification yielded the lowest accuracy of 0.6566.

Table 4 displays the classification results obtained using different sampling methods and classification algorithms, with feature selection conducted via the XGBoost feature selection method. Among these, the combination of SMOTE with XGBoost classification achieved the highest accuracy of 0.7563 while maintaining competitive average precision, recall, and F1 score values. These results underscore the importance of considering all performance metrics when evaluating the effectiveness of various sampling techniques and classification models.

Table 5 displays the classification results obtained using different sampling methods and classification algorithms, with feature selection conducted via the Gini index method. Among these, the XGBoost model consistently achieved the highest accuracy, attaining its peak performance of 0.749 with ROS. Notably, the No Sampling method yielded competitive results across various models, with the GB model achieving the highest recall score of 0.8028 and F1 score of 0.7722, respectively. Conversely, the ROS technique demonstrated relatively lower performance, particularly notable with the DT model, which achieved the lowest F1 score of 0.6414. The effectiveness of sampling methods varied across different algorithms, showcasing the importance of considering both the sampling strategy and the classification algorithm for optimal model performance.

We also determined the standard features selected by SHAP, XGBoost, and the Gini index, and these common features were used along with various sampling and classification methods to obtain the results presented in Table 6. Based on the data provided, it is evident that different combinations of sampling methods and classification models yield varying performance levels across different evaluation metrics. Notably, the No Sampling technique combined with the RF classifier consistently achieved the highest accuracy score of 0.7613, recall score of 0.789, and F1 score of 0.7806. This combination demonstrated strong precision, recall, and F1 scores, indicating robust performance across multiple metrics.

**Table 6:** Classification results using Common Features to explore various sampling and classification methods for optimal model performance

Sampling Method	Model	Accuracy	Avg Precision	Recall	F1 Score
ROS	SVM	0.719	0.8389	0.7054	0.7286
ROS	LG	0.6966	0.8009	0.7104	0.7158
ROS	KNN	0.6717	0.8127	0.6963	0.6941
ROS	ANNs	0.7214	0.8467	0.7331	0.7372
ROS	GB	0.7313	0.8428	0.7517	0.7503
ROS	XGB	0.7465	0.8669	0.7703	0.7641
ROS	RF	0.7389	0.8548	0.7377	0.7508
ROS	DT	0.649	0.6968	0.65	0.6637
No Sampling	SVM	0.7239	0.8405	0.7195	0.7362
No Sampling	LG	0.7015	0.8034	0.7102	0.7189
No Sampling	KNN	0.6916	0.817	0.7606	0.7259
No Sampling	ANNs	0.7238	0.8549	0.7517	0.7443
No Sampling	GB	0.7243	0.8435	0.7701	0.7491
No Sampling	XGB	0.744	0.862	0.7792	0.765
No Sampling	RF	0.7613	0.8626	0.789	0.7806
No Sampling	DT	0.6837	0.7192	0.742	0.7151
SMOTE	SVM	0.7313	0.8425	0.7284	0.7429
SMOTE	LG	0.6965	0.8033	0.7102	0.7151
SMOTE	KNN	0.6765	0.819	0.6957	0.697
SMOTE	ANNs	0.7313	0.8549	0.7474	0.7475
SMOTE	GB	0.7289	0.8573	0.7374	0.7451
SMOTE	XGB	0.7364	0.8632	0.7565	0.7562
SMOTE	RF	0.734	0.8636	0.7333	0.7472
SMOTE	DT	0.624	0.6646	0.6487	0.6463
RUS	SVM	0.7288	0.8356	0.7286	0.7429
RUS	LG	0.6966	0.8016	0.7197	0.7184
RUS	KNN	0.6766	0.8066	0.6773	0.6928
RUS	ANNs	0.7388	0.8479	0.737	0.7502
RUS	GB	0.7216	0.8374	0.7242	0.7362
RUS	XGB	0.7513	0.8659	0.7242	0.7581
RUS	RF	0.7463	0.8496	0.7284	0.7551
RUS	DT	0.6766	0.6856	0.7188	0.7022
NEAR-MISS	SVM	0.7287	0.8411	0.747	0.7469
NEAR-MISS	LG	0.7016	0.8019	0.7426	0.7281
NEAR-MISS	KNN	0.6941	0.8156	0.7558	0.726
NEAR-MISS	ANNs	0.7165	0.8386	0.7374	0.7356
NEAR-MISS	GB	0.7316	0.8487	0.7333	0.7462
NEAR-MISS	XGB	0.7465	0.8642	0.7335	0.7563
NEAR-MISS	RF	0.7462	0.8611	0.7281	0.7559
NEAR-MISS	DT	0.6787	0.6948	0.7102	0.7032

KNN = K-Nearest Neighbors, LR = Logistic Regression, SVM = Support Vector Machine, DT = Decision Tree, RF = Random Forest, GB = Gradient Boosting, XGB = XGBoost, and ANN = Artificial Neural Networks

Table 7 presents the classification results obtained using different sampling methods and algorithms without feature selection. The combination of RUS with GB classification achieved the highest accuracy score of 0.7565, demonstrating its effectiveness even without feature selection. On the other hand, the combination of ROS with DT classification resulted in the lowest accuracy score of 0.6443.

**Table 7:** Classification results using all 47 features (no feature selection) to explore various sampling and classification methods for optimal model performance

Sampling Method	Model	Accuracy	Avg Precision	Recall	F1 Score
ROS	SVM	0.6916	0.8076	0.7284	0.7164
ROS	LG	0.7016	0.8162	0.7190	0.7217
ROS	KNN	0.6743	0.7700	0.7468	0.7116
ROS	ANNs	0.7015	0.8210	0.7193	0.7196
ROS	GB	0.7290	0.8392	0.7329	0.7425
ROS	XGB	0.7315	0.8396	0.7465	0.7462
ROS	RF	0.7140	0.8162	0.7610	0.7407
ROS	DT	0.6443	0.6790	0.6498	0.6626
No Sampling	SVM	0.6993	0.8144	0.7147	0.7183
No Sampling	LG	0.6915	0.8135	0.7004	0.7086
No Sampling	KNN	0.6791	0.7768	0.8160	0.7323
No Sampling	ANNs	0.6891	0.8173	0.7422	0.7195
No Sampling	GB	0.7165	0.8552	0.7329	0.7357
No Sampling	XGB	0.7240	0.8423	0.7374	0.7415
No Sampling	RF	0.7315	0.8385	0.7890	0.7593
No Sampling	DT	0.6590	0.6997	0.7061	0.6910
SMOTE	SVM	0.6990	0.8101	0.7147	0.7162
SMOTE	LG	0.6941	0.8130	0.7190	0.7159
SMOTE	KNN	0.6691	0.7673	0.6864	0.6915
SMOTE	ANNs	0.6817	0.8221	0.7056	0.7029
SMOTE	GB	0.7118	0.8429	0.7149	0.7260
SMOTE	XGB	0.7265	0.8417	0.7190	0.7190
SMOTE	RF	0.7315	0.8349	0.7706	0.7532
SMOTE	DT	0.6716	0.7149	0.7201	0.7019
RUS	SVM	0.6915	0.8004	0.7097	0.7078
RUS	LG	0.6966	0.8095	0.7004	0.7129
RUS	KNN	0.6866	0.7622	0.7561	0.7234
RUS	ANNs	0.6540	0.7940	0.6589	0.6702
RUS	GB	0.7565	0.8543	0.7190	0.7377
RUS	XGB	0.7415	0.8478	0.7420	0.7546
RUS	RF	0.7189	0.8443	0.7193	0.7330
RUS	DT	0.6540	0.6905	0.6671	0.6725
NEAR-MISS	SVM	0.6993	0.8196	0.7009	0.7122
NEAR-MISS	LG	0.6891	0.8062	0.6911	0.7025
NEAR-MISS	KNN	0.6743	0.7732	0.7887	0.7227
NEAR-MISS	ANNs	0.6942	0.7859	0.7061	0.7112
NEAR-MISS	GB	0.7413	0.8438	0.7190	0.7484
NEAR-MISS	XGB	0.7465	0.8581	0.7329	0.7556
NEAR-MISS	RF	0.7539	0.8399	0.7383	0.7636
NEAR-MISS	DT	0.6691	0.6768	0.6552	0.6753

KNN = K-Nearest Neighbors, LR = Logistic Regression, SVM = Support Vector Machine, DT = Decision Tree, RF = Random Forest, GB = Gradient Boosting, XGB = XGBoost, and ANN = Artificial Neural Networks

The highest accuracy score of 0.7613 is achieved when employing the SHAP feature selection, NEAR-MISS sampling, and RF classification. Conversely, the best average precision score of 0.8847 was attained with the SHAP feature selection, No Sampling, and XGBoost classification. For recall, the combination of the Gini index Feature Selection, No Sampling, and GB classification outperformed others with a score of 0.8028. Lastly, the Common Features, No Sampling, and RF classification yielded the highest F1 score of 0.7806.

Tables 8, 9, and 12 showcase the mean performance metrics, including accuracy, average precision, recall, and F1 scores across various feature selection, sampling, and classification methods. Additionally, they highlight the best and worst values attained for each metric. Table 8 reports that the evaluation across multiple metrics reveals varying performance among the methods in the comparative analysis of SHAP, XGBoost, and the Gini index feature selection algorithms. SHAP demonstrated the highest mean accuracy score, 0.7199, followed closely by the Common Features (0.7142), with XGBoost trailing slightly behind at 0.7075. Moreover, SHAP and Common Features also outperformed the other methods, with the best accuracy score of 0.7613, indicating their superiority in achieving the highest classification accuracy in specific scenarios. Turning to precision, SHAP exhibited the highest mean precision score of 0.8260, with Common Features (0.8207), XGBoost (0.8141), and the Gini index (0.8082) following closely. In addition, SHAP also outperformed the other methods with the best precision score of 0.8847, indicating its superiority in achieving the highest classification precision in certain scenarios. When evaluating recall, SHAP demonstrated the highest mean recall score, 0.7393, with Common Features and XGBoost trailing closely behind with scores of 0.7295 and 0.7266, respectively. The Gini index (0.8028) achieved the best recall score, indicating its effectiveness in capturing more relevant instances. Finally, SHAP led in terms of F1 score, with the highest mean F1 score, 0.7390, followed closely by the Common Features (0.7323) and XGBoost (0.7275). Moreover, the best F1 score was achieved by Common Features (0.7806), emphasizing its satisfactory performance in achieving a balance between precision and recall.

**Table 8:** Comparative analysis of SHAP, XGBoost, and the Gini index feature selection algorithms with emphasis on common features

	SHAP	XGBoost	Gini index	Common Features
*Accuracy*				
Mean	**0.7199**	0.7075	0.7	0.7142
Best	**0.7613**	0.7563	0.749	0.7613
Worst	**0.6565**	0.6341	0.622	0.624
*Precision*				
Mean	**0.8260**	0.8141	0.8082	0.8207
Best	**0.8847**	0.8624	0.8599	0.8669
Worst	**0.6771**	0.6679	0.6508	0.6646
*Recall*				
Mean	**0.7393**	0.7266	0.7209	0.7295
Best	0.7981	0.7838	**0.8028**	0.789
Worst	**0.6835**	0.6502	0.6312	0.6487
*F1*				
Mean	**0.7390**	0.7275	0.72	0.7323
Best	0.7792	0.7747	0.7722	**0.7806**
Worst	**0.6813**	0.6537	0.6414	0.6463

We showed the model performance and compared with other machine learning model results to show that out model performes better. we also balanced the sample data and comapred with and without balancing

**Table 9:** Ranking SHAP, XGBoost, and the Gini index feature selection algorithms and common features in accuracy, precision, recall, and F1 scores

	SHAP	XGBoost	Gini index	Common Features
Accuracy	**1**	3	4	2
Precision	**1**	3	4	2
Recall	**1**	3	4	2
F1	**1**	3	4	2

We showed the model performance and compared with other machine learning model results to show that out model performes better. we also balanced the sample data and comapred with and without balancing

Table 9 ranks three feature selection algorithms, SHAP, XGBoost, and the Gini index, and their common features. SHAP consistently outperformed the other methods in this ranking, securing the top spot in every metric. Common Features also exhibited strong performance, ranking second, thereby underscoring the benefits of a unified feature set across different models. While valuable, XGBoost and the Gini index performed comparatively weaker in this analysis, ranking third and fourth, respectively.

Table 10 reports that when assessing mean accuracy across the different machine learning models, XGBoost performed the best with an accuracy score of 0.7429, followed by RF with an accuracy score of 0.7413. However, when considering the best accuracy, the RF model outperformed others with a score of 0.7613, indicating its superiority in certain instances. DT had the lowest mean accuracy score of 0.622, suggesting a comparatively weaker overall performance. Regarding average precision, XGBoost demonstrated the highest mean precision score of 0.8581, followed closely by GB, with a mean precision score of 0.8496. Yet again, XGBoost outshone the others with the best average precision score of 0.8847, while DT had the lowest mean average precision score of 0.6508. For recall, XGBoost led with a mean recall score of 0.7523, followed closely by RF with a mean recall score of 0.7486. GB attained the best recall score (0.8028), while DT had the lowest mean recall score (0.6312). Finally, XGBoost demonstrated the best mean F1 score of 0.7566, with RF achieving the highest F1 score of 0.7806. Conversely, DT exhibited the lowest mean F1 score of 0.6795.

**Table 10:** Performance comparison of eight ML models based on accuracy, average precision, recall, F1 score, and standard deviation

	**SVM**	**LR**	**KNN**	**ANNs**	**GB**	**XGB**	**RF**	**DT**
*Accuracy*								
Mean	0.7234	0.6961	0.6784	0.7176	0.7283	**0.7429**	0.7413	0.6555
Best	0.7438	0.7264	0.724	0.7388	0.7492	0.759	**0.7613**	0.6841
Worst	0.6941	0.6693	0.6346	0.6766	0.7015	0.7193	**0.724**	0.622
*Precision*								
Mean	0.8408	0.8111	0.7962	0.8472	0.8496	**0.8581**	0.8483	0.6866
Best	0.8561	0.8359	0.819	0.8683	0.8695	**0.8847**	0.8636	0.7192
Worst	0.8249	0.7912	0.7647	0.816	0.8336	0.8276	**0.8362**	0.6508
*Recall*								
Mean	0.7329	0.7104	0.7272	0.7341	0.7425	**0.7523**	0.7486	0.6846
Best	0.7697	0.7426	0.7929	0.7654	**0.8028**	0.7981	0.789	0.7424
Worst	0.6963	0.6779	0.6732	0.7052	0.6773	0.7006	**0.7199**	0.6312
*F1*								
Mean	0.7399	0.7156	0.7106	0.7352	0.745	**0.7566**	0.7553	0.6795
Best	0.7606	0.7405	0.7747	0.7522	0.7722	0.7792	**0.7806**	0.7151
Worst	0.7092	0.6884	0.6643	0.7005	0.7065	**0.7279**	0.7238	0.6414

KNN = K-Nearest Neighbors, LR = Logistic Regression, SVM = Support Vector Machine, DT = Decision Tree, RF = Random Forest, GB = Gradient Boosting, XGB = XGBoost, and ANN = Artificial Neural Networks

We showed the model performance and compared with other machine learning model results to show that out model performes better. we also balanced the sample data and comapred with and without balancing

Table 11 ranks eight ML models based on accuracy, average precision, recall, and F1 score. XGBoost was the top performer, demonstrating superior accuracy, high average precision, and a balanced precision-recall trade-off. GB, LR, and KNN ranked lower, indicating weaker performance. DT ranked lowest, indicating its limitations compared to other models.

**Table 11:** Ranking of ML models based on performance metrics: accuracy, precision, recall, and F1 score

	SVM	LR	KNN	ANNs	GB	XGB	RF	DT
Accuracy	3	6	7	4	5	**1**	2	8
Precision	3	6	7	2	4	**1**	5	8
Recall	3	8	4	5	2	**1**	6	7
F1	3	8	4	5	2	**1**	6	7

KNN = K-Nearest Neighbors, LR = Logistic Regression, SVM = Support Vector Machine, DT = Decision Tree, RF = Random Forest, GB = Gradient Boosting, XGB = XGBoost, and ANN = Artificial Neural Networks

We showed the model performance and compared with other machine learning model results to show that out model performes better. we also balanced the sample data and comapred with and without balancing

As shown in Table 12, the results indicate notable differences across different metrics in evaluating the performance of various sampling methods in machine learning. For accuracy, the sampling method with the highest mean accuracy was NO SAMPLING, closely followed by NEAR-MISS, with means of 0.7144 and 0.7128, respectively. Also, NO SAMPLING and NEAR-MISS demonstrated the highest accuracy in the best-case scenario, achieving a score of 0.7613. However, ROS had the lowest accuracy in the worst-case scenario, with a score of 0.622. For average precision, NO SAMPLING stood out for achieving the highest average precision in the best-case scenario with a score of 0.8874, maintaining its strong performance across all measures with the highest mean average precision of 0.8204. Nevertheless, SMOTE demonstrated notable average precision in the worst-case scenario with a score of 0.6508. Regarding recall, NO SAMPLING led in all aspects, with the highest mean performance score, 0.7507, and the highest recall score in the best-case (0.8028) and worst-case (0.6741) scenarios. Finally, analyzing F1 scores, NO SAMPLING maintained its dominance with the highest mean and best scores of 0.7384 and 0.7806, respectively. In contrast, NEAR-MISS achieved the highest F1 score in the worst-case scenario, with a score of 0.6703.

**Table 12:** Performance comparison of sampling methods and no-sampling in ML: accuracy, precision, recall, F1 score, and standard deviation

	SMOTE	ROS	NEAR-MISS	RUS	NO SAMPLING
*Accuracy*					
Mean	0.7082	0.7075	0.7128	0.7103	**0.7144**
Best	0.7563	0.7589	**0.7613**	0.7563	0.7613
Worst	0.624	0.622	**0.6419**	0.6393	0.6342
*Precision*					
Mean	0.8154	0.818	0.8195	0.8142	**0.8204**
Best	0.8636	0.8778	0.8695	0.8801	**0.8874**
Worst	0.6508	0.6515	**0.6825**	0.6727	0.6732
*Recall*					
Mean	0.7203	0.7206	0.7353	0.7187	**0.7507**
Best	0.7797	0.7797	0.7838	0.761	**0.8028**
Worst	0.6312	0.6372	0.674	0.6736	**0.6741**
*F1*					
Mean	0.7252	0.7249	0.7342	0.7268	**0.7384**
Best	0.7713	0.7735	0.7747	0.7704	**0.7806**
Worst	0.6463	0.6414	**0.6703**	0.6671	0.6655

We showed the model performance and compared with other machine learning model results to show that out model performes better. we also balanced the sample data and comapred with and without balancing

Table 13 ranks five sampling techniques—SMOTE, ROS, NEAR-MISS, RUS, and NO SAMPLING—based on accuracy, precision, recall, and F1 score. The NO SAMPLING approach, which does not involve sampling, consistently outperformed all others. NEAR-MISS ranked second, followed by RUS and SMOTE. ROS ranked lowest, indicating weaker performance. The NO SAMPLING technique consistently outperformed all others.

**Table 13:** Comparative ranking of sampling techniques for ML: SMOTE, ROS, Near-Miss, RUS, and No Sampling

	SMOTE	ROS	NEAR-MISS	RUS	NO SAMPLING
Accuracy	3	4	2	5	1
Precision	3	4	2	5	1
Recall	4	5	3	2	1
F1	4	5	3	2	1

Based on the comprehensive analysis across multiple tables, it is evident that SHAP emerged as the preferred feature selection method, consistently showcasing substantial accuracy, precision, recall, and F1 score performance. With respect to sampling methods, NEAR-MISS consistently yielded efficient results, particularly in conjunction with XGBoost classification. In terms of classification models, XGBoost stood out as the top performer, demonstrating superior performance across various metrics. Therefore, the combination of SHAP feature selection, the NEAR-MISS method, and XGBoost classification appeared to be the most effective at achieving high performance across different evaluation criteria.

**Fig. 3:**
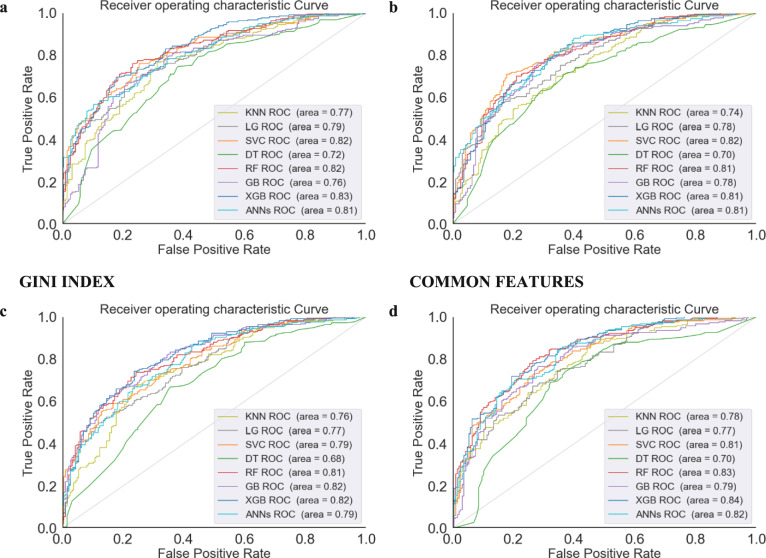
Performance of ML classifiers by feature selection method: AUC scores for SHAP, XGBOOST, GINI-INDEX, and Common Features

**Fig. 4:**
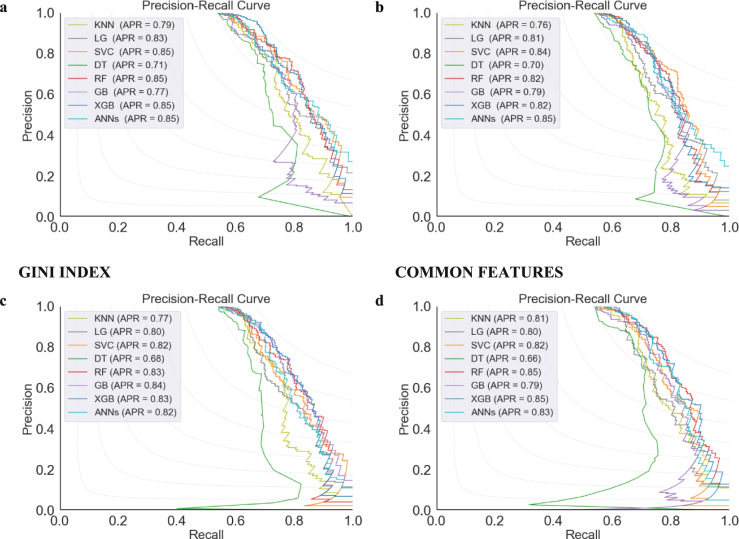
Performance of ML classifiers by feature selection method: APR scores for SHAP, XGBOOST, GINI-INDEX, and Common Features

Figure 3 represents the Area under the Curve (AUC) under three distinct feature selection methods, SHAP, XGBoost, and the GINI-INDEX, and a merger of common features identified by these three methods. SHAP performed consistently well, with top classifiers such as XGBoost, RF, and SVC achieving an AUC of 0.83, 0.82, and 0.82, respectively. RF and SVC had slightly lower AUC for their best-performing classifier (XGB at 0.83), suggesting a minor shortfall in capturing AD progression complexity. XGBoost feature selection compared well with SVC, achieving the highest AUC at 0.82. Combining common features resulted in similar AUCs, with ANNs at 0.82, XGB at 0.84, and RF at 0.83, showing that a consensus approach in feature selection was effective. Overall, SHAP and the common feature selection methods were marginally superior, with SHAP's interpretability and the robust biomarker identification of the common features method enhancing classifier performance. This emphasizes the importance of proper feature selection in improving AD progression predictive models and the quality of clinical prognostic tools. Moreover, Fig. 4 illustrates that the SHAP feature selection method showed high effectiveness, with RF, ANNs, SVC, and XGBoost achieving an Average Precision (APR) of 0.85, indicating precise feature identification. LG slightly reduced precision for some classifiers but maintained an APR of 0.83, suggesting its features fit them well. The GINI-INDEX provided strong performance, especially for GB, which achieved an APR of 0.84, highlighting its ability to capture predictive biomarkers. Classifiers using common features from SHAP, XGBoost, and the GINI-INDEX enhanced performance significantly, with XGB and RF achieving an APR of 0.85 and ANNs an APR of 0.83. The study suggests that while a consensus on feature selection methods creates a stable set of predictors, combining the unique attributes of each method may yield the best performance, as individual methods may reveal additional complexities.

**Fig. 5:**
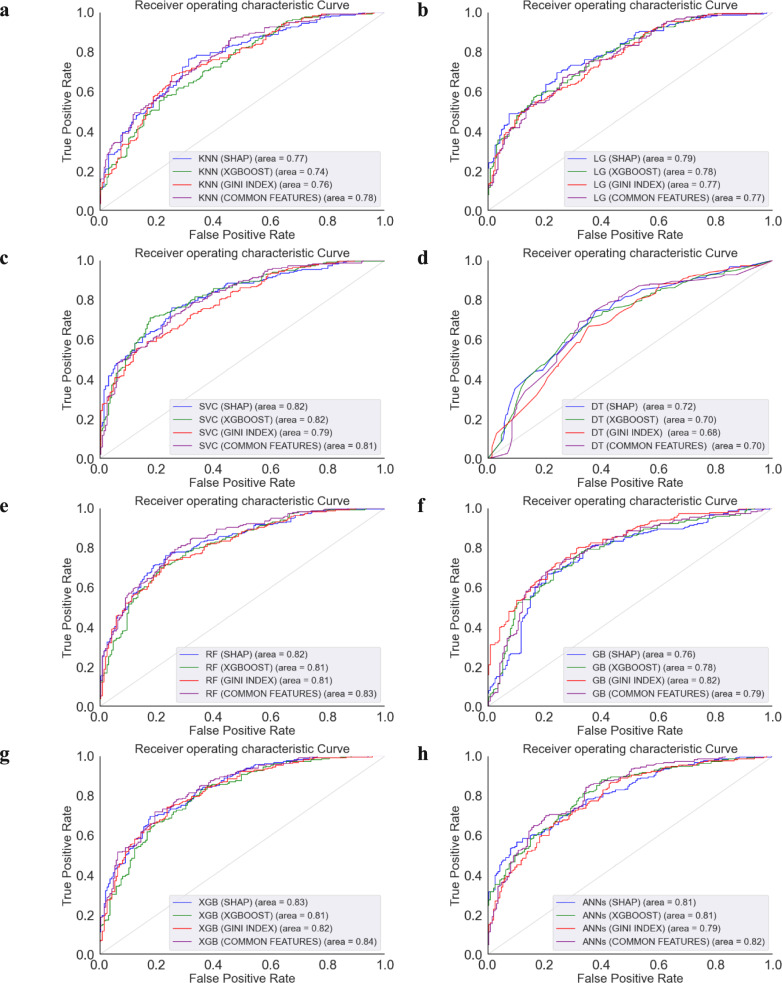
ROC curve analysis highlighting ML model performances for predicting progression from sMCI to pMCI

Figure 5 demonstrates that in ML models of AUC for predicting pMCI vs. sMCI, AUC reveals notable distinctions in model performance. The models evaluated included KNN, LG, SVM, DT, RF, GB, XGBoost, and ANNs. The receiver-operating characteristic (ROC) curves demonstrated that all models surpassed the baseline performance of random guessing, with AUC values ranging from 0.68 to 0.84. Notably, the XGBoost model emerged as the most proficient, exhibiting an AUC of 0.84, indicative of its superior capability to correctly classify the progression to MCI. With an AUC of 0.83, RF was ranked as the second most effective model. Although slightly behind the XGBoost in performance, the RF models demonstrated a strong predictive power, suggesting their valuable role in the diagnostic process of neurodegenerative diseases. Conversely, DT, with an AUC of 0.68, was identified as the least effective model in our study. The model rankings based on AUC values could serve as a preliminary guide for model selection.

**Fig. 6:**
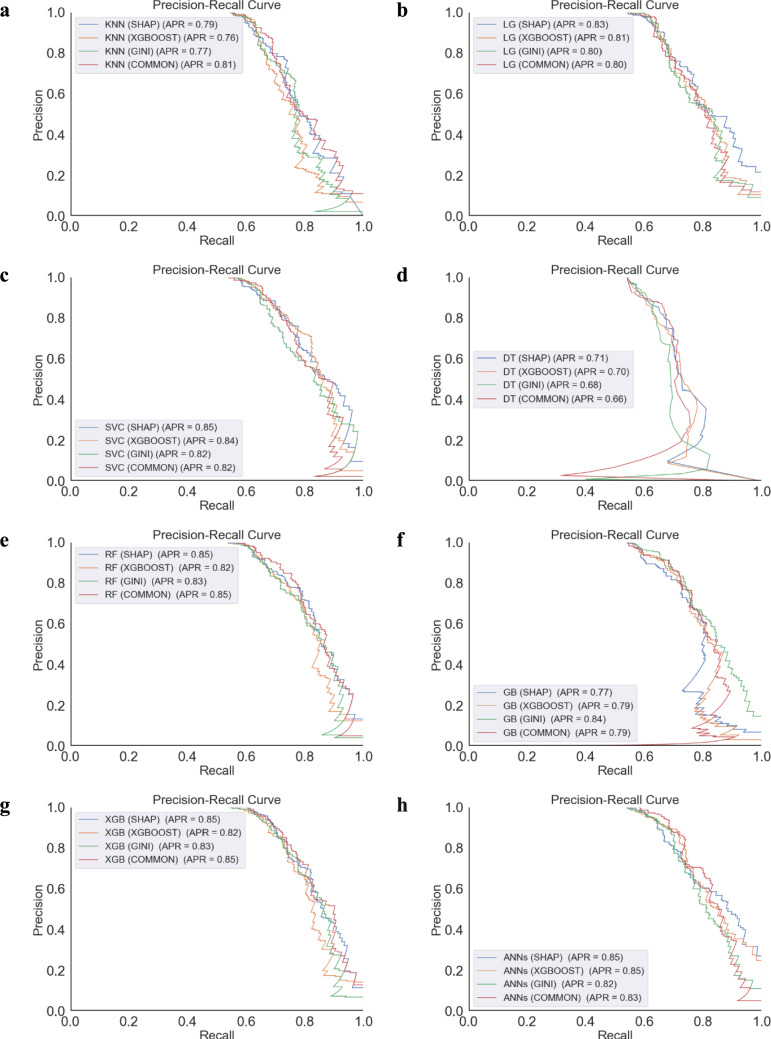
Precision-recall performance of ML models using multiple feature selection methods for progression from sMCI to pMCI

Figure 6 presents the precision-recall performance metrics of various ML models that predict the transition from sMCI to pMCI. The models assessed include KNN, LG, SVM, DT, RF, GB, XGBoost, and ANNs across different feature selection methods such as SHAP, XGBoost, the GINI-INDEX, and a combined approach using common features. The XGBoost model, across different feature selection methods, consistently showcased a high precision score of 0.85, reinforcing its position as the premier model for accurate classification of disease progression. The RF model also demonstrated a formidable precision score of 0.85, especially when informed by common feature selection methods. In contrast, the DT model registered a lower precision score of 0.68, particularly when common features are employed, underscoring its limitations in the face of complex diagnostic challenges. The numerical precision values reflected in the curves—highest for ANNs and RF and lowest for DT—serve as a quantitative guide for selecting and refining predictive models in clinical research settings.

## Discussion

In neurodegenerative research, particularly concerning the transition from sMCI to AD, the integrity and comparability of analytical outcomes are paramount. We employed a consistent ADNI database dataset to evaluate well-known classification algorithms. Such a homogenous application ensures that the variability in model performance is solely attributed to algorithmic efficacy and not confounded by disparate data sources. This methodological rigor provides a more accurate benchmark of the predictive capabilities of the algorithms and allows for direct comparisons, often obfuscated when different datasets are used. Conversely, using varied datasets across studies, while potentially offering broader validation of algorithmic robustness, may introduce variability that stems from dataset-specific characteristics rather than the intrinsic performance of the models. Differences in sample size, demographic distributions, biomarker baselines, and data quality contribute to this variability, leading to inconsistent findings and hindering the synthesis of results across studies. Our uniform approach to data use mitigates these issues and sets a precedent for future comparative studies in the field. By focusing on a single, well-characterized dataset, our work also capitalizes on the depth and quality of the data collected within ADNI, designed explicitly for AD research, and thus is more likely to contain relevant features for predicting AD progression. This advantage is critical in neuroimaging studies where selecting appropriate and sensitive biomarkers is crucial for early diagnosis and developing targeted interventions. Therefore, this study's findings offer compelling evidence for establishing a standardized dataset for ML applications in AD research. This can catalyze the development of robust, generalizable prediction models with clinical utility.

Our investigation into sampling methods, particularly the NEAR-MISS method, reveals some noteworthy insights. Although NEAR-MISS performed better among the sampling techniques evaluated, our results indicate that applying sampling methods generally negatively affects predictive performance compared to the no-sampling approach. This is a significant finding, as it suggests that avoiding sampling methods leads to higher performance metrics for our relatively balanced dataset (217 progressive MCI and 185 stable MCI).

The primary reason for exploring sampling methods was to conduct a comprehensive analysis and understand their potential impact on model performance. We hypothesized that even in a relatively balanced dataset, sampling methods might enhance the detection of minority class patterns and improve predictive accuracy. This hypothesis was based on the premise that sampling could provide a more balanced representation of the classes, leading to better model training and performance.

Compared to the Gini index and XGBoost, our results show that SHAP conferred significant strengths in achieving high prediction accuracy in progressing from MCI to AD.

SHAP provides interpretable and fair attributions across all features, making it valuable for clinical decision-making. The Gini index, commonly used in decision trees, measures the impurity reduction of each feature, highlighting features that partition patients into groups with different outcomes. XGBoost, an implementation of gradient-boosted decision trees, incorporates a built-in feature importance measure to help identify features most predictive of the outcome. The study identified several biomarkers, including neuroimaging indicators such as hippocampal volume and cortical thickness, cognitive test scores, and biological markers such as amyloid beta and tau protein levels. These features have been extensively documented as critical indicators of AD progression.

Understanding the role of individual features can significantly enhance model accuracy and reliability. The ability of SHAP to perform well on its own and highlight the significance of shared features underscores the utility of interpretability in feature selection. SHAP's performance in precision and recall demonstrates its capability to identify relevant instances accurately and with a high degree of reliability. This balance between precision and recall, captured through the F1 score, points to SHAP's effectiveness in providing a holistic understanding of model behavior. Thus, incorporating SHAP into the feature selection process enhances model interpretability and predictive accuracy, highlighting its value in advancing the development of more nuanced and effective predictive models. Its standout performance across multiple metrics demonstrates the importance of interpretability in feature selection, particularly in AD classification, which requires precise and reliable predictions. SHAP's insights enhance model improvement and provide a comprehensive understanding of model factors, enabling the creation of more effective and interpretable predictive tools.

## Conclusion

This study analyzes ML techniques for predicting the progression from MCI to AD using a unified dataset from the ADNI. Incorporating the Shapley value explanation (SHAP) and other feature selection methods, such as the Gini index and XGBoost, highlights the importance of biomarkers and enhances the accuracy and interpretability of predictive models in clinical settings. SHAP demonstrated superior precision and recall metrics performance, emphasizing the importance of interpretability in feature selection. Key biomarkers were identified through SHAP, underscoring the potential use of ML in early diagnosis and tailored treatment plans. The balanced approach to dataset sampling and comparison across multiple classification algorithms strengthened the validity of the findings. The study emphasizes the synergistic value of integrating various feature selection techniques to refine predictive modeling in AD and underscores the importance of interpretability in ML applications.

## Data Availability

The data are available for download on the ADNI website (https://adni.loni.usc.edu/data-samples/access-data/).
